# The differences in the consumption of proteins, fats and carbohydrates in the diet of pregnant women diagnosed with arterial hypertension or arterial hypertension and hypothyroidism

**DOI:** 10.1186/s12884-019-2711-y

**Published:** 2020-01-13

**Authors:** Wioletta Waksmańska, Rafał Bobiński, Izabela Ulman-Włodarz, Anna Pielesz

**Affiliations:** 1 Faculty of Health Sciences, University of Bielsko-Biala, ul Willowa 2, 43-300 Bielsko-Biala, Poland; 2 Faculty of Materials, Civil and Environmental Engineering, University of Bielsko-Biala, ul Willowa 2, 43-300 Bielsko-Biala, Poland

## Abstract

**Background:**

Excessive body weight induces the occurrence of arterial hypertension. The risk associated with irregularities during the perinatal period is increased in women with diagnosed hypothyroidism. Disorders of thyroid functions during pregnancy may cause higher body weight gains**.** The aim of this project was to determine the differences in the average daily intake of proteins, fats and carbohydrates in women with arterial hypertension and with hypothyroidism.

**Methods:**

The control group (Group I) included healthy women. In this group, no complications during the course of pregnancy were observed and the delivery was on the due date. Group II was comprised of patients with arterial hypertension. Group III included patients with arterial hypertension, who were diagnosed with hypothyroidism before pregnancy. The women’s eating habits and dietary composition were analyzed based on a dietary assessment.

**Results:**

Women with arterial hypertension (Group II) consumed the highest number of calories per day, while women with a normal pregnancy consumed the lowest amount of calories. The daily consumption of vegetable protein was similar in all study groups. The average daily consumption of fat, cholesterol and carbohydrates was the highest among women with diagnosed arterial hypertension. Women with arterial hypertension and hypothyroidism more frequently gave birth before the 38th week of pregnancy. The average daily intake of Arginine, Lysine, Methionine and Tryptophan was lower in the group of women with a normal pregnancy than in the two other groups.

**Conclusions:**

Excessive calorie intake causing significant body weight gain fostered the occurrence of arterial hypertension during pregnancy.

## Background

The healthy lifestyle of a pregnant woman is an important factor that affects her child’s health. Numerous publications provide recommendations concerning a healthy diet, while advertisements and TV programs both emphasize the role of proper selection of food products containing the relevant nutrients. However, for a person lacking knowledge on balancing a proper diet, the selection of food products alone may not be sufficient.

An improperly composed diet, with either a deficiency and an excess of nutrients, may adversely affect the course of pregnancy and labor [1]. The diet of a pregnant woman should contain all the nutrients necessary for the mother and the child, with particular regard to the caloric value.

A mother’s pre-pregnancy obesity is strongly associated with the risk of excessive body weight in her offspring, and an increased risk of nervous system defects in the fetus [1–3].

A woman’s excessive body weight gain during pregnancy also adversely affects the fetus and the mother and obesity occurring during pregnancy has become a serious public health issue. Excessive weight gains during pregnancy cause obesity in the child, increase the risk of cardiovascular diseases, increase systolic and diastolic blood pressure, and result in insulin resistance. In the perinatal period, the incidence of preterm birth and Cesarean section increase, likewise with perinatal mortality. It is estimated that in Western countries, obesity affects up to 30% of pregnant women, whereas 40% of women gain excessive body weight during pregnancy [1].

Excessive body weight induces the occurrence of arterial hypertension the, frequency of which increases in the case of an improperly balanced diet due to, amongst others, excessive intake of sodium. Arterial hypertension is a common complication during pregnancy, and is connected with an increased risk of health complications in both the mother and her offspring.

The risk associated with irregularities during the perinatal period is additionally increased in women with diagnosed hypothyroidism. Disorders of thyroid functions during pregnancy may cause higher body weight gains, while at the same time this weight gain may lead to changes in thyroid functions [4].

An important element in the prevention of complications during pregnancy and the perinatal period is a properly balanced diet, which can be achieved once the most common dietary mistakes made by pregnant women are recognized. Such knowledge will significantly influence proper meal planning and allow nurses to undertake actions to promote a healthy lifestyle.

### Aim

The aim of this project was to determine the differences in the average daily intake of proteins, fats and carbohydrates amongst women with arterial hypertension recognized during pregnancy and amongst women with additional hypothyroidism.

## Research and method

The research was conducted in the Maternity Hospital in Tychy among a group of women with a single pregnancy in the period from the fourth quarter of 2016 until the second quarter of 2017. The study examined the protein, fat and carbohydrate content in the diet of the pregnant women. The analysis included women with arterial hypertension diagnosed during pregnancy. Women with arterial hypertension recognized before pregnancy were excluded from the research.

Women with hypothyroidism diagnosed before pregnancy were qualified to the research group. Women with other chronic diseases were excluded from the study (two women), as were all women with infections (five women) and those who smoked cigarettes (two women). Before the study was conducted, a special meeting took place in order to obtain consent for the research to be conducted and to explain the method of filling in the questionnaire. The research included 182 women.

The patients participating in the research were divided into three groups. Group II was comprised of patients with arterial hypertension recognized in the I trimester of pregnancy (95 patients). Group III included patients with arterial hypertension diagnosed in the I trimester of pregnancy and those diagnosed with hypothyroidism before pregnancy (39 patients). The control group (Group I) included healthy women (45 patients). In this group, no complications during the course of pregnancy were observed and the delivery was on the due date (Table 1). However, the analysis did not include three women due to insufficient data in the questionnaire. Altogether, 179 women aged 20–39 were involved in the study. The average age of the tested subjects was 29.

**Table 1:** Presentation of the studied groups of women

Hbd	Group I	Cesarean section		Group II	Cesarean section		Group III	Cesarean section	
		*n*	% of n		*n*	% of n		*n*	% of n
34	–	–	–	–	–		8	8	100%
35	–	–	–	1	1	100%	4	4	100%
36	–	–	–	3	2	67%	8	6	75%
37	–	–	–	5	3	60%	10	7	70%
Total before 38 Hbd	–	–	–	9	6	67%	30	25	83%
38	10	1	10%	35	21	60%	6	4	67%
39	17	2	12%	25	10	40%	3	3	100%
40	7	1	14%	20	7	35%	0	–	–
41	11	2	18%	6	3	50%	0	–	–
Total before 38 Hbd	45	6	13%	86	41	48%	9	7	78%
Total	45	6	13%	95	47	50%	39	29	74%

Gestational age was calculated according to Naegele’s rule. Information on the women’s course of pregnancy came from the Pregnancy Record, i.e. the document maintained by the gynecologist. All information concerning the birth and postpartum period was taken from documents kept by the hospital.

The women’s eating habits and dietary composition were analyzed based on a dietary assessment. Portion sizes were verified using “The Album of Photographs of Food Products and Dishes”. The album contains photos presenting meal portions of various sizes accompanied by data on the portions’ size expressed in grams. This allows the researcher to precisely determine the amount of daily food intake [5]. The questionnaire was completed by participants with the help of the person conducting the research.

The questionnaire allowed the researchers to determine the daily consumption of each particular dietary component (proteins, carbohydrates, fats), as well as the mother’s calorie consumption over a one-month period. The DIETA FAO program, which includes data on 1067 typical Polish dishes or food products, was used to estimate the quantity of the aforementioned components. The testing procedure was described in detail in an earlier publication [6].

The energy and nutrient requirements were determined for pregnant women weighing 65 kg, and the water intake required (pure water and water contained in food) was established to be 2.5 l/day. The average intake values obtained were compared with United States Department of Agriculture dietary guidelines and with nutrition standards for the Polish population [7, 8]. BMI values were calculated according to body weight before pregnancy and body weight in the last week of pregnancy.

### Statistical analysis

In order to optimize the conclusions of the analysis, appropriate methods and statistical analysis tools were applied. In forecasting variable values, significant predictors (strong scales) and regression models of numerous variables were used. The achieved level of the analyses was *p* < 0.05. Variable distribution normality was checked by means of the Shapiro-Wilk’s test. The results of the tests clearly demonstrated that the variables had a normal distribution. Before commencing the statistical analysis, the person conducting the study checked if the data was complete.

Analysis of variance (ANOVA) was applied in order to test the hypotheses on the lack of differences between the values of particular tested variables defining inter-group relations. All relations between the tested variables were determined with the use of correlation analysis and the Pearson coefficient. Relations between the compared test results were estimated with the use of one-factor ridge regression analysis. When simplified, the biometric regression model adopted the following general form:

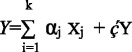



In order to determine the occurrence of statistically significant differences in particular groups of variables, the post-hoc RIR Tukey test was conducted.

The calculations were done by means of Statistica software (StatSoft Poland) and Microsoft Office Excel spreadsheets (Microsoft Poland).

The study was approved by the Bioethics Review Board of Bielsko-Biala (No: 2016/02/11/4) in accordance with the Helsinki Declaration.

## Results

In Group I, a Cesarean section was performed on 13% of the women, and none of the women in this group gave birth before the 38th week of pregnancy. Every second woman in Group II had a Cesarean section, whereas in Group III, Cesarean section was performed more often - in 74% of the women. The women in Group III (with arterial hypertension and hypothyroidism) more frequently gave birth before the 38th week of pregnancy (Table 1).

Nine women were excluded from the study: seven women with chronic diseases and infections, and two women who smoked cigarettes. Also, three other women were excluded due to insufficient data in the questionnaire.

Women with arterial hypertension (Group II) consumed the highest number of calories per day, while women with a normal pregnancy consumed the lowest amount of calories. The highest amount of proteins was found in the diet of women with arterial hypertension (126.17 g/day), and a similar amount of proteins was consumed by women with hypertension and hypothyroidism (123.94 g/day). The lowest amount of proteins was consumed by women with a normal pregnancy (98.39 g/day). Consumption of animal protein was at a similar level – women with hypertension had an intake of 88.87 g/day, while women with hypertension and hypothyroidism - 88.49 g/day. The lowest amount of animal protein was consumed by women with a normal pregnancy (67.23 g/day). The daily consumption of vegetable protein was similar in all groups: women with a normal pregnancy – 31.15 g/day, women with arterial hypertension – 35.44 g/day, women with arterial hypertension and hypothyroidism – 34.88 g/day. The highest amount of fat, cholesterol and carbohydrates consumed daily was by women with diagnosed arterial hypertension (fat 160.5 g/day, cholesterol 723.01 mg/day, carbohydrates 367.63 g/day), the lowest amount – by women with a normal pregnancy (fat 89.98 g/day, cholesterol 431.99 mg/day, carbohydrates 298.24 g/day). The average daily consumption of fat, cholesterol and carbohydrates was the highest among women with diagnosed arterial hypertension and the lowest among women with a normal pregnancy. The fat consumption standard was not exceeded in any of the analyzed groups of women, unlike the daily carbohydrate intake, which was exceeded in all groups. The daily standard of cholesterol consumption was not specified, however, the results obtained during the analysis indicate that the consumption is very high in the tested groups. In the case of the average daily intake of water, the situation was similar. The group of women with arterial hypertension consumed a statistically significant larger amount of water than healthy women. The average daily intake of Arginine, Lysine, Methionine and Tryptophan was lower in the group of women with a normal pregnancy than in the two other groups (Table 2). Analysis of variance (ANOVA) showed the inter-group relations of the analyzed variables (Table 3). The analysis of particular variables demonstrated statistically significant differences with regard to the week of pregnancy (HBD), while the post-hoc RIR Tukey test showed statistically significant differences between values differentiating particular groups (Table 4). In the case of the energy variable, the post-hoc RIR Tukey test demonstrated statistically significant differences between all the groups analyzed. No statistically significant differences between Group II and Group III were observed in the average daily consumption of water (Table 5). Differences in the average daily consumption of protein (total protein, animal and vegetable protein) and amino acids were statistically significant in the comparison between healthy women (Group I), the group of women with arterial hypertension (Group II) and those with arterial hypertension and hypothyroidism (Group III) (Tables 6 and 7). In the case of the variable for the average daily consumption of fat, the post-hoc RIR Tukey test showed statistically significant differences between the differentiating values. In the case of cholesterol intake in the groups analyzed, no statistically significant differences between Group II and Group III were observed. Statistically significant differences between the values differentiating Group I from Group II and III were observed in the case of carbohydrate consumption (Table 8). There was also a statistically significant difference between the BMI value before and during pregnancy (Table 9).

**Table 2:** Presentation of standard deviation and average daily intake of selected nutrients in analysed groups of women

Studied variable and unit	Group I		Group II		Group III		Daily norm of average consumption for pregnant woman
	*M*	S	*M*	S	*M*	S	
Hbd	39,400	1136	38,726	1198	36,282	1572	
Birth weight (g)	3272,444	341,029	3408,316	389,505	2590,641	520,008	2501 g
BMI before pregnancy	22,158	1,56	23,236	2,11	22,781	1,98	
BMI in pregnancy	26,318	3327	28,741	4119	27,544	3944	–
Energy (kcal)	2750,813	391,073	3328,366	230,906	3195,795	283,874	2500–3000 kcal
Water (ml)	2485,613	664,411	3072,348	359,442	3146,142	429,572	2500 ml
Total protein (g)	98,394	15,529	126,174	12,647	123,938	11,499	0.98 g/kg body weight
Animal protein (g)	67,238	15,307	88,887	13,194	88,492	13,824	–
Vegetable protein (g)	31,156	7182	35,446	4651	34,886	6167	–
Fats (g)	89,984	20,832	160,505	22,803	148,448	24,564	70–115 g
Total carbohydrates (g)	298,236	71,092	367,629	39,706	362,116	48,239	175 g
Cholesterol (mg)	431,989	154,903	723,015	174,960	707,824	139,483	Not determined
Arginine (mg)	5143,114	932,426	6551,987	1008,743	6497,063	961,915	–
Lysine (mg)	6732,208	1343,944	8540,414	948,030	8625,783	938,273	–
Methionine (mg)	2362,959	400,933	3052,673	345,839	3030,839	371,696	–
Tryptophan (mg)	1289,453	232,741	1580,673	229,270	1574,964	199,852	–

*kcal* kilocalories, *g* gram, *mg* milligram, *μg* microgram, *ml* mililitr

**Table 3:** Statistically significant differences of the tested parameters between Group I, II and III demonstrated by the analysis of variance (ANOVA)

Variable	F
Hbd	70,816*
BMI	15,748*
Energy	61,061*
Total protein	71,530*
Animal protein	40,520*
Vegetable protein	8917*
Arginine	34,063*
Lysine	50,548*
Methionine	58,793*
Tryptophan	28,119*
Fats	150,334*
Cholesterol	52,235*
Total carbohydrates	29,840*
Water	28,768*

*F* distribution, *statistical significance below 0.005

**Table 4:** Statistically significant differences in the analysed groups of women referring to the end of pregnancy week

Studied variable Hbd	Group I	Group II	Group III
	39,4	38,7	36,2
Group I		0,009728*	0,000022*
Group II	0,009728*		0,000022*
Group III	0,000022*	0,000022*	

*statistical significance below 0.005

**Table 5:** Statistically significant differences in the analysed groups of women referring to average daily consumption of calories and water

Studied variable/ average	Group I		Group II		Group III	
	Energy	Water	Energy	Water	Energy	Water
	2750,81	2485,61	3328,36	3072,34	3195,79	3146,14
Group I			0,000022*	0,000022*	0,000022*	0,000022*
Group II	0,000022*	0,000022*			0,042891*	0,685,141
Group III	0,000022*	0,000022*	0,042891*	0,685,141		

*statistical significance below 0.05

**Table 6:** Statistically significant differences in the analysed groups of women referring to average daily consumption of proteins

Studied variable/ average	Group I			Group II			Group III		
	Total protein	Animal protein	Vegetable protein	Total protein	Animal protein	Vegetable protein	Total protein	Animal protein	Vegetable protein
	98,39	67,23	31,15	126,17	88,88	35,44	123,93	88,49	34,88
Group I				0,000022*	0,000022*	0,000117*	0,000022*	0,000022*	0,008040*
Group II	0,000022*	0,000022*	0,000117*				0,646,283	0,987,744	0,863,584
Group III	0,000022*	0,000022*	0,008040*	0,646,283	0,987,744	0,863,584			

*statistical significance below 0.005

**Table 7:** Statistically significant differences in the analysed groups of women referring to average daily consumption of Arginine, Lysine, Methionine and Tryptophan

Studied variable/ average	Group I				Group II				Group III			
	Arginine	Lysine	Methionine	Tryptophan	Arginine	Lysine	Methionine	Tryptophan	Arginine	Lysine	Methionine	Tryptophan
	5143,1	6732,2	2363,0	1289,5	6552,0	8540,4	3052,7	1580,7	6497,1	8625,8	3030,8	1575,0
Group I					0,00022*	0,00022*	0,00022*	0,00022*	0,00022*	0,00022*	0,00022*	0,00022*
Group II	0,00022*	0,00022*	0,00022*	0,00022*					0,953,266	0,905,737	0,947,183	0,990,163
Group III	0,00022*	0,00022*	0,00022*	0,00022*	0,953,266	0,905,737	0,947,183	0,990,163				

*statistical significance below 0.005

**Table 8:** Statistically significant differences in the analysed groups of women referring to average daily consumption of Fats, Cholesterol and Carbohydrates

Studied variable/ average	Group I			Group II			Group III		
	Fats	Cholesterol	Carbohydrates	Fats	Cholesterol	Carbohydrates	Fats	Cholesterol	Carbohydrates
	89,98	431,98	298,23	160,50	723,01	367,62	148,44	707,82	362,11
Group I				0,000022*	0,000022*	0,009728*	0,000022*	0,000022*	0,000022*
Group II	0,000022*	0,000022*	0,000022*				0,014614**	0,875,969	0,837,339
Group III	0,000022*	0,000022*	0,000022*	0,014614**	0,875,969	0,837,339			

*statistical significance below 0.005, **statistical significance below 0.01

**Table 9:** Correlation of BMI values in the studied groups of women before and during pregnancy

Group	BMI		Correlation coefficient value
	Before pregnancy	In pregnancy	
Group I	22,158	26,318	0,97,258*
Group II	23,236	28,741	0,91,551*
Group III	22,781	27,544	0,88,769*

*statistical significance below 0.05

## Discussion

The nutritional status of pregnant women has an important influence on the development of the fetus. Nutrient deficiency may occur both in women with excessive weight gain and in underweight women.

Improper weight gain during pregnancy may be caused, amongst others, by a too low or too high calorific value of meals with regard to the body’s needs. Excessive weight gains during pregnancy have a negative impact on the course of pregnancy and its conclusion. Being overweight or obese during pregnancy increases the risk of giving birth to a child with macrosomy, associated with a higher risk of performing a Cesarean section and increased risk of perinatal complications. In those women who developed obesity during pregnancy, preeclampsia may occur [9, 10].

Analysis of the author’s own material showed that the BMI value before pregnancy was within the normal range in all the women. The highest BMI increase was observed in the group of women with hypertension. The same group of women also consumed the most calories during the day. A relatively lower increase in BMI value was observed in the group of women who, in addition to hypertension, were also affected by hypothyroidism. In these two groups of women, relatively frequent cases of Cesarean section were reported, over half of which were performed before the 38th week of pregnancy. The age of the pregnant women was not found to affect the frequency of performing a Cesarean section – the mean age of the study participants was 29 years.

The risk of giving birth to a child with macrosomy is lower in women with proper body weight or those who are slightly overweight, hence Cesarean section is less frequently performed. In these women, there are the risks of giving birth to a child with low birth weight and the occurrence of premature birth [9, 10]. In the authors’ studies, the lowest mean body weight was observed in women with hypothyroidism and hypertension. No relationship was found between the BMI value in pregnant women and the birth weight of the child.

The World Health Organization has expressed its opinion that a rate of Cesarean sections higher than 10–15% in a given region is not justified. Despite WHO recommendations, the percentage of Cesarean sections continues to increase [11].

The authors’ own research showed that women with a normal pregnancy had the lowest increase in BMI value during pregnancy, while at the same time, Cesarean section was performed in the case of 13% of the women. In the group of women with complicated pregnancy, hypertension and hypothyroidism, the rate was as high as 74%, out of which over 60% were performed before week 38.

This is confirmed in research on women with a single pregnancy by Matalon et al. who stated that the percentage of Cesarean sections in women with hypothyroidism was higher than in healthy women. At the same time, in the case of hypothyroidism, Cesarean sections were more frequent at earlier stages of pregnancy [12]. No studies were found in the literature on the frequency of Cesarean sections and the occurrence of excessive weight in women with hypothyroidism and hypertension.

According to Männistö and co-authors, thyroid disease affects 4% of pregnant women, hypothyroidism being the most common. According to research conducted in Delhi, hypothyroidism was present in 13.13% of pregnant women. Inadequately treated thyroid diseases increase the risk of anemia, miscarriage, premature birth and hypertension. Untreated hypothyroidism during pregnancy may slow down a child’s mental development [12, 13].

Hypertension during pregnancy is induced by endothelial dysfunction of the blood vessels, resulting from differences in the amounts of the substance that dilates vessels and the substance that constricts vessels. Nitrogen oxide, which inhibits secretion of renin and endothelin, is responsible for decreasing the tension of blood vessels. Nitrogen oxide is formed through a reaction synthesized by endothelial enzymes in the presence of arginine. Arginine in the serum of pregnant women with hypotrophy of the fetus attains lower values than in the case of healthy women.

According to Pardej and co-authors, the daily requirement for arginine is 1.88 g. In the case of a daily intake of protein at the level of 30–60 g, the content of arginine in the diet is 3–6 g [14]. In the authors’ own studies, the group of women with hypertension and hypothyroidism consumed approximately 120 g of protein, which amounted to a relatively high consumption of arginine, i.e. 6–12 g per day.

It is important that a pregnant woman’s diet contains an adequate amount of nutrients, while maintaining the appropriate caloric value of meals.

An essential part of the diet is complete protein, which fulfills an important function in the body of a pregnant woman as it provides the necessary amino acids for the fetus. Protein deficiency during pregnancy may lead to inhibition of fetus growth. An excess of proteins causes an excessive increase in body mass. Excessive consumption of animal protein may lead to kidney dysfunction and, as a result, to the development of kidney stones [14].

The authors’ own research found that in all of the groups analyzed, the women consumed an excessive amount of protein, and that, in the case of women with hypertension and hypothyroidism, the recommended daily intake was exceeded by more than double. The daily consumption of protein was similar. The high consumption of protein in the group of women with hypertension caused weight gain, and consequently a change in BMI value. Such a high increase in body weight may have had an influence on the development of abnormal blood pressure values.

The nutrients consumed by women affect intestinal microflora which, in turn, impact on human health and incidence of disease. This is particularly important as bacteria are transferred during childbirth and initiate the process of colonization in infants [15].

Excessive fat intake and a high consumption of sugar are particularly negative in the diet of a pregnant woman. They can lead to macrosomy and thus to the need to perform a Cesarean section, and may also cause hypoglycemia in the fetus [2].

According to the authors’ own research, the average daily consumption of fat and carbohydrates was the highest among women with diagnosed arterial hypertension and the lowest among women with a normal pregnancy. The literature does not specify a daily standard for cholesterol consumption, however, the results obtained during the analysis indicate that the consumption in the tested groups is very high.

The studied sample was not representative at a national level, which is a limitation of the research. Another limitation was the lack of respondents’ objectivity in assessing the amount of food consumed.

However, the respondents voluntarily participated in the research and it may be assumed that they are interested in issues of health and proper nutrition, which is a positive aspect of the research. Moreover, the analysis of dietary patterns will enable dietary interventions to be adapted to make them more appropriate for pregnant women living in a particular region.

## Conclusions

The average daily consumption of calories, proteins, fats and carbohydrates was statistically significantly higher in the groups of women with arterial hypertension and with arterial hypertension and hypothyroidism than in the group of women with a normal pregnancy.

Excessive calorie intake causing significant body weight gain fostered the occurrence of arterial hypertension during pregnancy. The incidence of Cesarean sections in the groups of women diagnosed with arterial hypertension or arterial hypertension and hypothyroidism was significantly higher than in the group of women with a normal pregnancy.

## Data Availability

The datasets analyzed during the current study are not publicly available due to the need to protect sensitive data; however, they are available from the corresponding author on reasonable request.

## Abbreviations

ANOVA: Analysis of variance
BMI: Body Mass Index
g: Gram
HBD: Hebdomas graviditatis
mg: Milligram
TV: Television
